# Spiral phase infrared imaging with undetected photons using a visible wavelength spatial light modulator

**DOI:** 10.1038/s41598-026-43775-3

**Published:** 2026-03-19

**Authors:** Osian Wolley, Emma Pearce, Simon P. Mekhail, Thomas Gregory, Miles J. Padgett

**Affiliations:** 1 School of Physics and Astronomy, University of Glasgow, Glasgow, G12 8QQ UK; 2 Institut für Physik, Humboldt-Universität zu Berlin, 12489 Berlin, Germany

**Keywords:** Optics and photonics, Physics

## Abstract

Imaging with undetected photons utilises induced coherence between downconverted photon pairs in a nonlinear interferometer to record an image of an object using infrared probe photons whilst only detecting photons at an easier-to-detect visible wavelength that never interacts with the object. We show that the induced coherence between photon pairs also allows for manipulation of the Fourier components of infrared light from the object, by implementing phase masks on a spatial light modulator placed in the visible beam. By applying a spiral phase mask to the visible beam we demonstrate a well-known Fourier filtering technique, namely spiral phase-contrast imaging of an object placed in the infrared beam, giving omnidirectional edge enhancement of the image. This approach could also be applied to other Fourier filtering microscopy techniques, such as dark-field and phase-contrast microscopy using undetected photons.

## Introduction

Nonlinear interferometry enables the measurement of a sample illuminated in the infrared (IR) while only detecting the interference of position-correlated visible photons that have never interacted with the sample, often referred to as imaging with undetected photons^1^. Due to this ability to probe a sample at IR wavelengths—where detector technology poses a challenge in terms of cost, efficiency, and noise performance—whilst detecting with silicon-based detectors, sensing with undetected photons has been demonstrated for gas sensing^2^, spectroscopy^3–5^ and optical coherence tomography^6,7^. For wide-field imaging, the advantage is clear, since IR detector arrays have lower sensitivity and are typically lower resolution than their visible counterparts. One area of interest is the application of undetected photons to microscopy, with demonstrations of mid-infrared microscopy employing either momentum anti-correlations^8^ or position correlations^9^. With these methods, it is possible to obtain both intensity and phase images of an object in the infrared beam, using either phase-shifting or off-axis holography^2,8,10–14^.

In conventional microscopy, spatial light modulators (SLMs) are commonly placed in the Fourier plane of a 4*f* system to control the spatial frequency components of the imaging light. This configuration allows for the point spread function (PSF) to be engineered, enabling various techniques to enhance imaging performance^15^. These approaches include correcting aberrations^16,17^, extending the depth of focus for 3D imaging^18,19^, and providing versatile programmable Fourier filters for contrast methods such as dark-field^20^, Zernike phase contrast^21^, and spiral phase contrast^22,23^. However, SLMs are typically based on liquid crystal devices which, although effective in the visible and near-IR regions of the spectrum, are not effective in the mid-IR.

Integrating SLMs into imaging with undetected photons in this way opens up the possibility to apply these techniques to IR imaging using visible detectors. Crucially, the interference effect that underpins imaging with undetected photons depends on the phase of both the detected visible light and the imaging IR light, thus making it possible to use a visible-wavelength SLM to perform the PSF engineering. The IR light therefore interacts with neither the SLM nor the camera.

### Fourier filtering and spiral phase contrast

There are several well-known methods for improving the image contrast of phase objects by manipulating the Fourier components of light transmitted through the object. For example, in Zernike phase contrast^21^, shifting the phase of the zeroth-order Fourier component by a quarter wavelength with respect to higher-order components, gives an image with enhanced contrast of the phase variations of the sample. In dark field microscopy^20^, the zeroth order component can simply be blocked, resulting in phase details within the image appearing bright against a dark background. Another type of filter is a spiral phase filter, with a helical phase proportional to $e^{i \ell \varphi }$, where $\ell$ is the helical index and $\varphi$ the angular polar coordinate^24^. These spiral filters are associated with the creation of light beams carrying orbital angular momentum^25^. When $\ell =1$ a phase mask is created that has a phase varying from $0-2\pi$ around a central axis with a phase singularity located in the centre. If the spiral phase mask is located in the Fourier plane of the object, the image of a phase object appears dark except for the edges of the object, in an effect known as spiral phase contrast^22,23^. While a spiral phase plate also provides a way to create an $\ell =1$ spiral phase filter, SLMs have been demonstrated as an effective means of generating a spiral phase filter, and have the advantage of being able to switch between different imaging modalities (e.g. dark-field or phase-contrast) without the need to switch physical optics^15,26,27^. The spiral phase pattern displayed on the SLM can be combined with a blazed diffraction grating, also displayed on the SLM, to produce a forked grating hologram. This diffracts the incoming light, where the $1^{\text {st}}$ is used for imaging and the undiffracted zeroth order can be blocked, allowing for improved modulation of the imaging light. It is the spiral phase filter which we implement to demonstrate the ability to use an SLM as a Fourier filter in imaging with undetected photons.

A full mathematical description of spiral phase imaging is provided in^15^, which is summarised here. The spiral phase filter, in combination with a Fourier transform lens, transforms a collimated beam to a focused helically-phased beam, with a ring-shaped intensity beam. As a result, the PSF of the imaging system has a ring-shaped intensity pattern, with an azimuthally varying phase between $0-2\pi$. In the object plane, each object pixel becomes one of these doughnut rings weighted by the complex object transmission function for that pixel, which is then integrated over the whole area. The resulting intensity of the pixel in the image plane is then mainly determined by the complex value of neighbouring pixels, such that regions with little structure will appear dark as neighbouring pixels will be $\pi$ out of phase due to the spiral phase topology of each ring and destructively interfere. In the case of a change in amplitude however, the intensity of neighbouring rings is different across the amplitude variation, and thus there is not complete destructive interference and these regions appear brighter than their surroundings. In the case of a phase change, the neighbouring pixels across the jump in phase are no longer $\pi$ out of phase and complete destructive interference does not occur, and the edge at the jump will appear brighter. In these cases, any phase edges present in the image will appear brighter than their surroundings. This property underlies the omni-directional edge enhancement behind spiral phase contrast imaging and is therefore easily translated into imaging with undetected photons because of its phase-sensitive nature.

The use of spatial light modulators for imaging with undetected photons remains a relatively unexplored topic. An SLM was first used to create a phase object for a demonstration of a classical equivalent for imaging with undetected photons^28^. Another recent experiment used an SLM to perform synthetic off-axis holography with undetected photons, by displaying a linear phase gradient on an SLM in place of a tilted mirror to obtain tilt fringes at the detector^14^. In both of these demonstrations, the SLM is in the image plane of the object; whereas to control the PSF, the SLM needs to be placed in the Fourier plane. Another approach to visible imaging of IR illumination is up-conversion, using sum-frequency generation between the IR image and a pump laser to up-convert the image to a visible wavelength. Spiral phase plates have been used to shape the pump laser, enabling edge enhancement and dark-field up-conversion imaging^29,30^. However, this approach requires high-power pump lasers and dedicated IR sources which increase the cost, complexity, and noise, especially for wide-field imaging^8^. Further, the sample is exposed to much more IR light than is subsequently used for the visible image retrieval.

In this work, we demonstrate the ability to use a visible-wavelength SLM to manipulate the Fourier components of IR light at an object in an imaging with undetected photons system. By displaying a spiral phase pattern on the SLM in the path of the visible photons, omnidirectional edge enhancement is seen when imaging an object in the infrared path, despite the infrared light interacting with neither the SLM nor the camera. This proof-of-concept suggests that other enhanced imaging modalities, such as dark-field or phase-contrast, can also be realised with undetected photons.

## Results

**Fig. 1:**
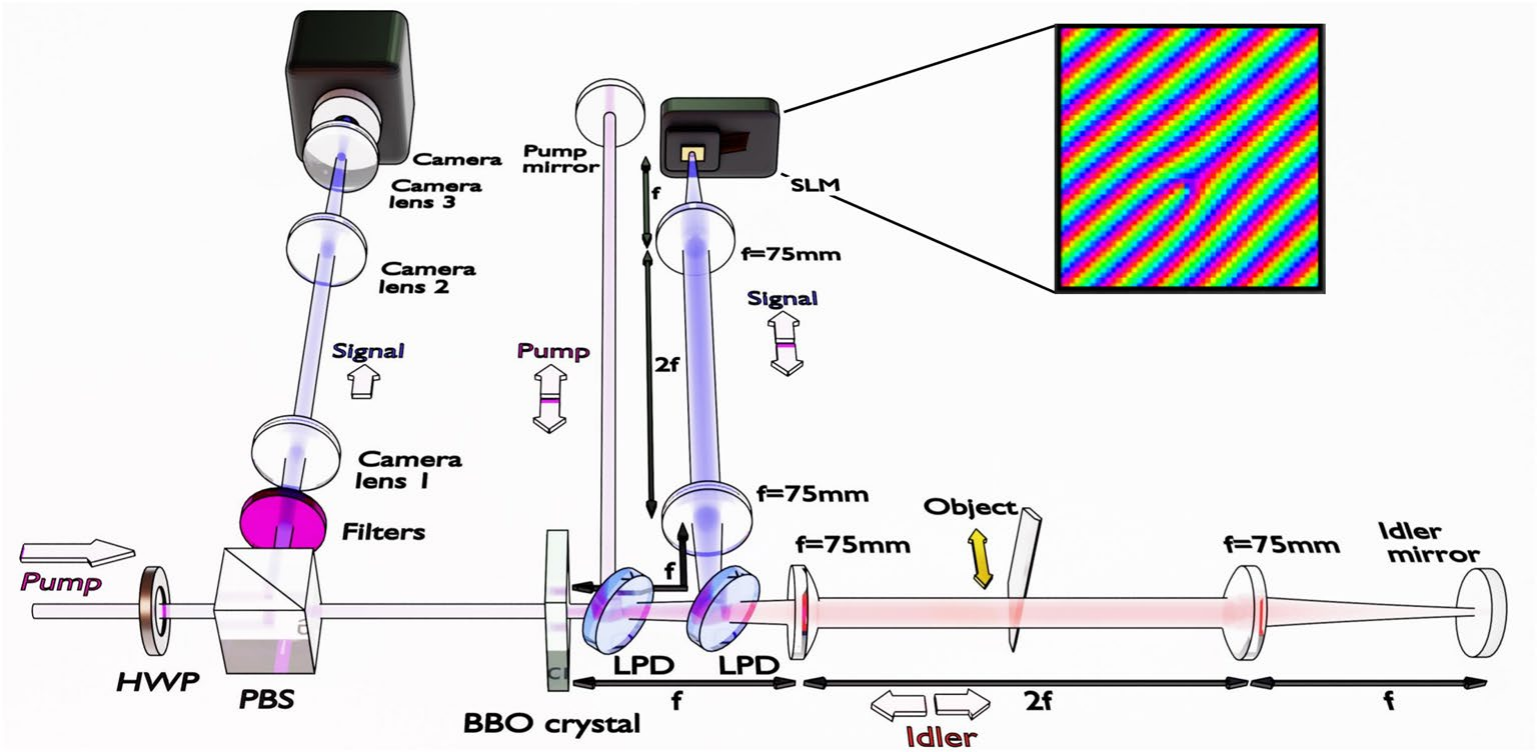
Diagram of the experimental setup for Fourier-plane filtering in imaging with undetected photons. A 355 nm pump laser passes through a half-wave plate (HWP) and polarising beamsplitter (PBS) and generates non-degenerate photon pairs (460 nm signal, 1555 nm idler) in a BBO crystal. Long-pass dichroic mirrors (LPD) first separate the pump from the photon pairs, then separate signal and idler photons. Lenses (*f* = 75 mm) form matched 4*f* imaging systems in the signal and idler paths to preserve spatial correlations. In the signal path, a spatial light modulator (SLM) is located at 4*f* from the crystal. In the idler path, the object is located at 2*f* from the crystal, placing the object in a Fourier plane of both the crystal and the SLM. After the second pass through the crystal, the signal is separated by the PBS towards the camera, where camera lenses 1, 2, and 3 image the object. Inset: example SLM pattern for spiral phase contrast.

Figure 1 shows the experimental setup used in this work (see “Methods” for details). Non-degenerate photon pairs, comprising a visible signal photon (460 nm) and an IR idler photon (1555 nm), can be generated by a pump laser either during its first pass through the nonlinear crystal or its return pass after reflection. When the signal, idler, and pump modes are carefully overlapped, any information which distinguishes between pairs generated in the first pass or return pass is erased, leading to interference in the signal photon counts seen at the camera. Introducing an object in the path of the first-pass IR idler photons breaks this indistinguishability, which is observed as changes to the visible interference pattern. Phase shifts introduced by the object appear as shifts in the interference fringes, while the IR transmittance of the object corresponds to the fringe visibility. In contrast to ghost imaging, coincidence detection with the idler is unnecessary; the interference depends solely on the potential to obtain the “which-pass” information.

Within a nonlinear interferometer designed for imaging, the choice of lens system in the signal and idler paths of the interferometer is limited by the need to maintain spatial correlations between signal and idler so full-field imaging can be performed. Imaging with undetected photons has been demonstrated utilising both momentum anti-correlations and position correlations. In practice, this means choosing a lens system such that the object and camera are placed in a Fourier or image plane of the crystal, and that the lens system performs the same transform in the signal and idler paths of the interferometer, to maintain the spatial correlations. The inclusion of an SLM places an additional constraint; in order to shape the Fourier components of the light from the object, the SLM must be placed in the signal path such that it sits in the plane corresponding to the Fourier plane of the object in the idler beam. We used the folded Michelson style design of the SU(1,1) interferometer which has become common in imaging with undetected photons, placing a 4*f* imaging system in both the signal and idler paths. This allows for the signal mirror to be replaced with an SLM, and an object to be placed in a Fourier plane of both the SLM and the down-conversion crystal. A series of lenses are then used to image the Fourier plane of the crystal and object onto the camera. This imaging system maintains spatial correlations between photon pairs and places the SLM in a Fourier plane of the object without double-passing the SLM.

An interesting consequence of the Michelson-style nonlinear interferometer used here is that the object is double passed. Usually in imaging with undetected photons schemes this is not an issue as the object is placed on, or close to, the idler mirror such that the two images overlap. However, in this system, two images of the object are formed in two halves of the field-of-view. Only one half of the field-of-view was used for reconstruction, which effectively halves the number of spatial modes available for imaging. This issue could be alleviated by adopting a Mach-Zehnder style design of the nonlinear interferometer, as was used in the initial demonstration of imaging with undetected photons, although with an increase in system complexity^1^.

**Fig. 2:**
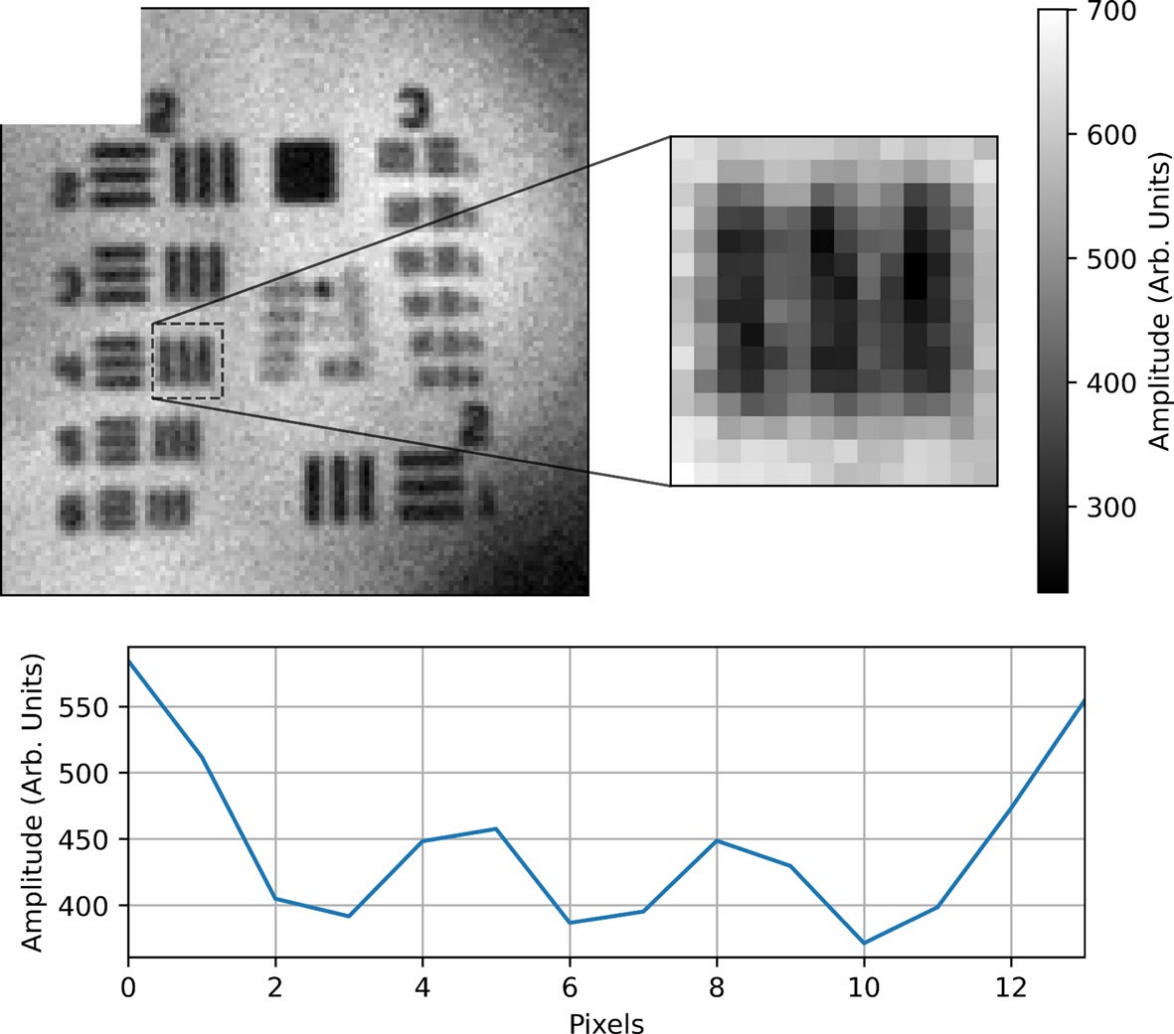
Determination of the resolution of the imaging system with a USAF target. The smallest resolvable element of the USAF test target was determined to be element 4 of group 2, which is shown enlarged and a horizontal cross section of the intensity of the enlarged region is also shown. Element 4 of group 2 corresponds to a spatial frequency of $5.66 \,\,\text {lp\,mm}^{-1}$.

To assess the resolution of the imaging system, a USAF resolution test target was imaged as shown in Fig. 2. It was determined from Fig. 2 that the smallest resolvable feature was element 4 of group 2. This gives an estimate of the resolution of the system as $5.66\,\text {lp mm}^{-1}$ (line pairs per millimetre), or a resolvable feature size of $88\,\mu \text {m}$. With the object placed in the far-field of the downconversion of the crystal, the theoretical value of the resolution is given by

1 $$\begin{aligned} \delta x_{corr} = \frac{\sqrt{2\ln {2}}f \lambda _i}{\pi w_p }, \end{aligned}$$

where *f* is the focal length of the lens used to image the far-field of the crystal onto the object, $\lambda _i$ the idler wavelength, $w_p$ the pump beam waist^8^. For this experiment, this gives the resolution of the system as $\sim 87\,\mu \text {m}$, agreeing well with the measured smallest feature size of $88\,\mu \text {m}$. Similarly, allowing for the bandwidth of the down-converted idler photons the field of view was modelled^31^, giving the theoretical value of $22.0\,\text {mm}$. The actual field of view was measured to be smaller than this at $14.6\,\text {mm}$. The discrepancy between these values is likely due to a slight mismatch in the centre frequencies of the bandpass filter before the camera. Using the measured values of the resolution and the field of view, the number of spatial modes and hence the number of independent pixels in the image can be estimated as $\sim (100)^2$.

Ideally, the interferometer should be aligned on-axis and the path length adjusted until the zero phase delay point is reached, and hence uniform constructive interference or destructive interference should be seen across the whole field of view. However, we observed residual interference fringes likely arising from wavefront aberrations in the optical system. Phase-stepping reconstruction methods^32^ were used to form the final amplitude images in order to minimise the effect of any phase instability. Single-frame, off-axis holography methods for undetected photons^12,13^ could not be used here due to the 4*f* lens system in the signal and idler paths, meaning a tilt of either the idler mirror or SLM would not produce a change in angle of the interfering beams in the far-field of the crystal.

**Fig. 3:**
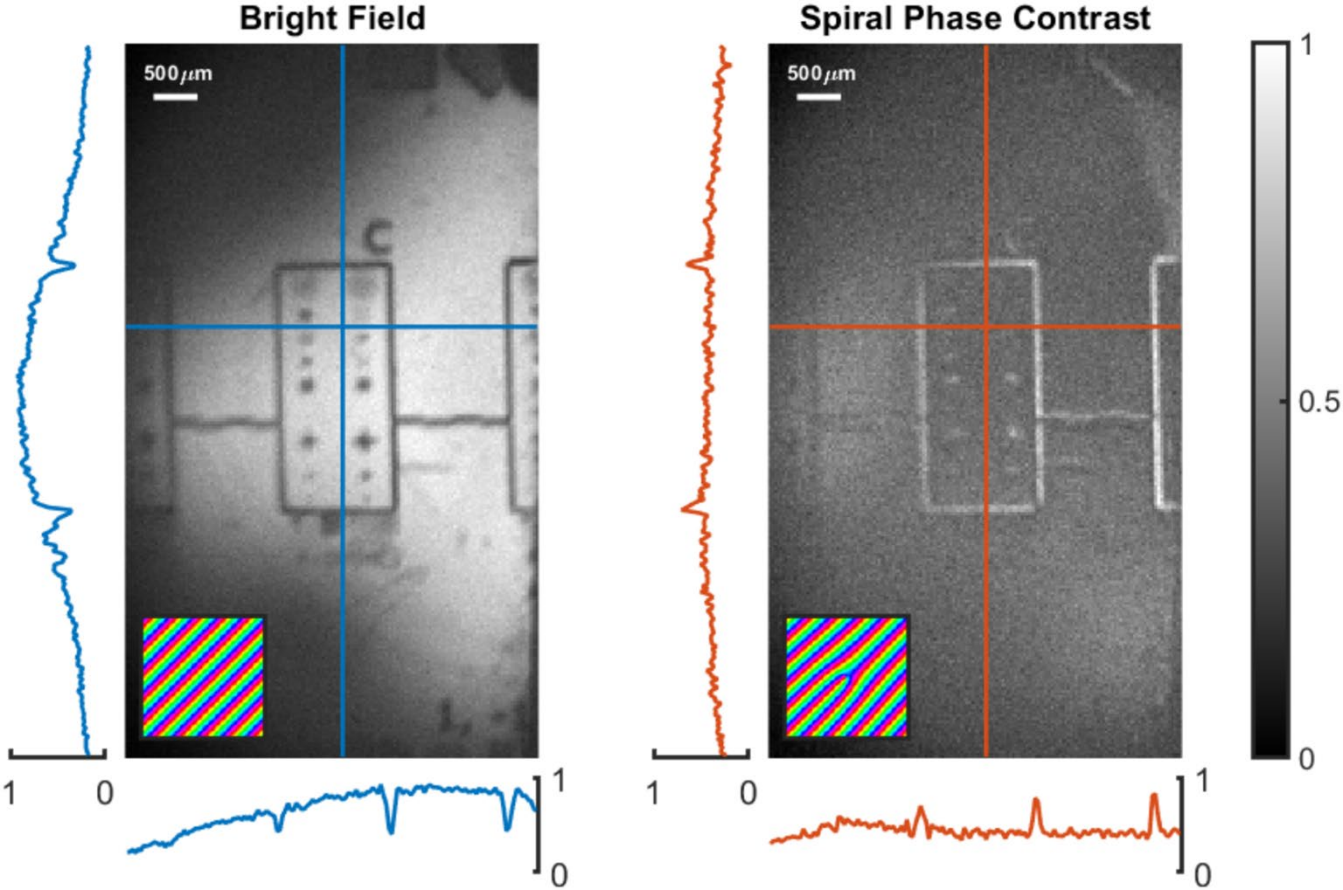
Bright field and spiral phase contrast images of a phase object acquired with the system. Each image is the average of 100 intensity images reconstructed from phase stepping. Insets show the pattern displayed on the SLM for the generation of each image. Typical amplitude profiles are shown, corresponding to the horizontal and vertical lines in the main images.

To demonstrate the omni-directional edge enhancement, we used a photo-resist mask as a phase object. Figure 3 shows the results of applying a spiral phase mask to the grating displayed on the SLM whilst imaging. Each image is the average of 100 intensity images, reconstructed by phase stepping. Without the spiral phase mask applied, a bright-field image of the object is obtained, where the edges of the etchings appear dark against a bright background. This is reversed for in the case of the spiral phase point spread function. The spiral phase PSF works to suppress slow changes across the field of view, flattening the background compared to the bright field imaging. Edges of features therefore stand out as bright peaks against a more uniform dark background. In these results we see the intensity of the spiral phase filtered image corresponds to $\sim 70\%$ of the total bright field image intensity.

## Methods

A collimated 100 mW, 355 nm pump beam is prepared in polarisation by a half-wave plate and polarising beamsplitter before pumping a 1 mm thick BBO crystal. The crystal is cut for type-I non-degenerate downconversion, producing signal and idler photon pairs at 460 nm and 1555 nm respectively. The signal and idler photon pairs are separated into two paths using a long-pass dichroic mirror. Signal lenses 1 and 2 ($f\,=\,75$ mm) are used to image the crystal to the plane of the SLM. A grating is displayed on the SLM and, by tilting the SLM, the 1st diffraction order is then reflected back towards the crystal. Idler lens 1 and 2 ($f\,=\,75$ mm) similarly image the crystal onto the idler mirror, with the object placed in the far-field of the crystal. A long-pass dichroic mirror placed after the crystal separates the pump beam from the signal and idler photons, and the pump mirror reflects the pump back towards the crystal. The pump mirror is also a distance of 4*f* from the crystal such that pump, signal, and idler paths are matched. This step is necessary to protect the SLM from damage by the UV pump laser light. The second pass of the pump beam through the crystal generates a second pair of signal and idler photons, where overlap of the idler modes results in induced coherence between the two signal beams. It is worthwhile to note that the interference occurs not between light in the two “arms” of the interferometer, but rather between photons pairs which may be created in the first or second pass of the non-linear crystal. To observe this interference, the camera is placed in the far-field of the crystal and the object is re-imaged by camera lenses 1, 2, and 3.

## Discussion

Here we show the use of a spatial light modulator within an imaging with undetected photons scheme to obtain images showing an omnidirectional edge-enhancement effect. The full edge enhancement could be observed by phase stepping and averaging over several of these phase cycles which mitigates phase instabilities. This phase stepping is easily performed using the SLM, removing the need for moving parts in the interferometer. The limited stability of the current system is not intrinsic to the technique, and edge-enhancement does not inherently require phase-stepped measurements. With improved interferometric stability, single-frame edge-enhanced imaging should be achievable, enabling video-rate operation, as previously demonstrated in imaging with undetected photons^12^.

It is suggested here that switching to a Mach-Zehnder interferometer layout with two separated nonlinear crystals could help address both the issue of low fringe visibility and phase instability; the pump could be separated with a sturdier element to reduce phase instability introduced by the dichroic mirror, and loss compensation could be implemented with the use of polarisation optics in the pump beam^33^. The Michelson geometry employed here results in a double pass of the object, as the idler is reflected such that it returns to the crystal. As noted earlier, this produces two spatially separated images in the field-of-view, effectively halving the usable imaging area. A Mach–Zehnder configuration would also alleviate this issue, since the idler would be transmitted through the object only once; this would also reduce attenuation, which is beneficial when imaging low-transmission samples such as microfluidic devices^34^.

The diameter of the signal beam at the SLM is determined by the diameter of the pump beam at the crystal, since the pump effectively defines the aperture within which the SPDC process can occur; this aperture is then imaged onto the SLM. The SLM used in this experiment has a pixel size of 20 $\mu$m, corresponding to approximately 50 pixels across the diameter of the signal beam, or about 2,500 pixels in total covering the beam area. From the spatial-mode estimates given above, the signal contains roughly $\sim$10,000 spatial modes. Because the number of spatial modes exceeds the number of available SLM pixels, the SLM cannot independently address all modes. This likely limits the fidelity with which the phase mask can be implemented, and may account for the relatively low contrast observed in our spiral phase-contrast images. Using an SLM with smaller pixels could help alleviate this issue, and potentially lead to demonstrations of other imaging modalities.

In conclusion, our demonstration highlights the ability to use an SLM operating at visible wavelengths to perform point spread function engineering of infrared wavelengths in imaging with undetected photons. We have applied this ability to spiral phase imaging by introducing an SLM in the visible path at a Fourier plane of an object which is itself in the IR path. This gives omnidirectional edge enhancement of the image despite the IR illumination light never interacting with the camera or the SLM. We anticipate that this proof-of-concept will lead to further exploration of image enhancement techniques using SLMs in imaging with undetected photons, particularly at mid-IR wavelengths where neither SLMs nor efficient, low-cost cameras are available.

## Data Availability

All data used in this study are available from the corresponding author upon reasonable request.

## References

[1] Lemos, G. B. et al. Quantum imaging with undetected photons. *Nature* **512**, 409–412 (2014). 25164751 10.1038/nature13586

[2] Dong, J. et al. Methane sensing via unbalanced nonlinear interferometry using a CMOS camera and undetected mid-infrared light. *Appl. Phys. Lett.* **126** (2025).

[3] Kalashnikov, D. A., Paterova, A. V., Kulik, S. P. & Krivitsky, L. A. Infrared spectroscopy with visible light. *Nat. Photon.* **10**, 98–101 (2016).

[4] Lindner, C., Wolf, S., Kiessling, J. & Kühnemann, F. Fourier transform infrared spectroscopy with visible light. *Opt. Exp.* **28**, 4426–4432 (2020). 10.1364/OE.382351 32121679

[5] Paterova, A. V., Toa, Z. S. D., Yang, H. & Krivitsky, L. A. Broadband quantum spectroscopy at the fingerprint mid-infrared region. *ACS Photon.* **9**, 2151–2159 (2022).

[6] Paterova, A. V., Yang, H., An, C., Kalashnikov, D. A. & Krivitsky, L. A. Tunable optical coherence tomography in the infrared range using visible photons. *Quantum Sci. Technol.* **3**, 025008 (2018).

[7] Vanselow, A. et al. Frequency-domain optical coherence tomography with undetected mid-infrared photons. *Optica* **7**, 1729 (2020).

[8] Kviatkovsky, I., Chrzanowski, H. M., Avery, E. G., Bartolomaeus, H. & Ramelow, S. Microscopy with undetected photons in the mid-infrared. *Sci. Adv.* **6**, eabd0264 (2020). 10.1126/sciadv.abd0264 PMC10763735 33055168

[9] Kviatkovsky, I., Chrzanowski, H. M. & Ramelow, S. Mid-infrared microscopy via position correlations of undetected photons. *Opt. Exp.* **30**, 5916–5925 (2022). 10.1364/OE.440534 35209543

[10] Paterova, A. V., Maniam, S. M., Yang, H., Grenci, G. & Krivitsky, L. A. Hyperspectral infrared microscopy with visible light. *Sci. Adv.* **6**, eabd0460 (2020). 10.1126/sciadv.abd0460 PMC7608807 33127685

[11] Töpfer, S. et al. Quantum holography with undetected light. *Sci. Adv.* **8**, eabl4301 (2022). 10.1126/sciadv.abl4301 PMC8759747 35030021

[12] Pearce, E. et al. Single-frame transmission and phase imaging using off-axis holography with undetected photons. *Sci. Rep.* **14**, 16008 (2024). 38992022 10.1038/s41598-024-66233-4 PMC11239902

[13] León-Torres, J. R. et al. Off-axis holographic imaging with undetected light. *Opt. Exp.* **32**, 35449–35461 (2024). 10.1364/OE.528724 40514905

[14] Töpfer, S. et al. Synthetic quantum holography with undetected light. *Opt. Quantum* **3**, 129–136 (2025).

[15] Maurer, C., Jesacher, A., Bernet, S. & Ritsch-Marte, M. What spatial light modulators can do for optical microscopy. *Laser Photon. Rev.* **5**, 81–101 (2011).

[16] Jesacher, A. & Booth, M. J. Parallel direct laser writing in three dimensions with spatially dependent aberration correction. *Opt. Exp.* **18**, 21090–21099 (2010). 10.1364/OE.18.021090 20941005

[17] Cameron, P. et al. Adaptive optical imaging with entangled photons. *Science* **383**, 1142–1148 (2024). 38452085 10.1126/science.adk7825

[18] Maurer, C., Khan, S., Fassl, S., Bernet, S. & Ritsch-Marte, M. Depth of field multiplexing in microscopy. *Opt. Exp.* **18**, 3023–3034 (2010). 10.1364/OE.18.003023 20174133

[19] Quirin, S., Peterka, D. S. & Yuste, R. Instantaneous three-dimensional sensing using spatial light modulator illumination with extended depth of field imaging. *Opt. Exp.* **21**, 16007–16021 (2013). 10.1364/OE.21.016007 PMC3971059 23842387

[20] Murphy, D. B. & Davidson, M. W. Phase contrast microscopy and darkfield microscopy. In *Fundamentals of Light Microscopy and Electronic Imaging*. Chap. 7. 115–133 (Wiley, 2012).

[21] Zernike, F. The concept of degree of coherence and its application to optical problems. *Physica* **5**, 785–795 (1938).

[22] Fürhapter, S., Jesacher, A., Bernet, S. & Ritsch-Marte, M. Spiral phase contrast imaging in microscopy. *Opt. Exp.* **13**, 689–694 (2005). 10.1364/opex.13.000689 19494929

[23] Bernet, S., Jesacher, A., Fürhapter, S., Maurer, C. & Ritsch-Marte, M. Quantitative imaging of complex samples by spiral phase contrast microscopy. *Opt. Exp.* **14**, 3792 (2006). 10.1364/oe.14.003792 19516527

[24] Oron, R., Davidson, N., Friesem, A. A. & Hasman, E. Chapter 6 - Transverse mode shaping and selection in laser resonators. In *Progress in Optics* (Wolf, E. Ed.). *Progress in Optics*. Vol. 42. 325–386 (Elsevier, 2001).

[25] Beijersbergen, M., Coerwinkel, R., Kristensen, M. & Woerdman, J. Helical-wavefront laser beams produced with a spiral phaseplate. *Opt. Commun.* **112**, 321–327 (1994).

[26] Maurer, C., Jesacher, A., Fürhapter, S., Bernet, S. & Ritsch-Marte, M. Tailoring of arbitrary optical vector beams. *New J. Phys.* **9**, 78 (2007).

[27] Jesacher, A., Fürhapter, S., Bernet, S. & Ritsch-Marte, M. Shadow effects in spiral phase contrast microscopy. *Phys. Rev. Lett.* **94**, 233902 (2005). 16090473 10.1103/PhysRevLett.94.233902

[28] Cardoso, A. C. et al. Classical imaging with undetected light. *Phys. Rev. A* **97** (2018).

[29] Junaid, S., Tidemand-Lichtenberg, P., Pedersen, C. & Rodrigo, P. J. Upconversion dark-field imaging with extended field of view at video frame rate. *Appl. Opt.* **59**, 2157–2164 (2020). 32225742 10.1364/AO.384502

[30] Qiu, X., Li, F., Zhang, W., Zhu, Z. & Chen, L. Spiral phase contrast imaging in nonlinear optics: Seeing phase objects using invisible illumination. *Optica* **5**, 208–212 (2018).

[31] Boeuf, N. et al. Calculating characteristics of noncollinear phase matching in uniaxial and biaxial crystals. *Opt. Eng.* **39**, 1016 (2000).

[32] Yamaguchi, I. & Zhang, T. Phase-shifting digital holography. *Opt. Lett.* **22**, 1268–1270 (1997). 18185816 10.1364/ol.22.001268

[33] Gemmell, N. R. et al. Loss-compensated and enhanced midinfrared interaction-free sensing with undetected photons. *Phys. Rev. Appl.* **19**, 054019 (2023).

[34] Suryana, M. et al. Infrared imaging with visible light in microfluidic devices: The water absorption barrier. *Analyst* **150**, 405–413 (2025). 39692693 10.1039/d4an01201a

